# Evaluating the Impact of the Dementia Care in Hospitals Program (DCHP) on Hospital-Acquired Complications: Study Protocol

**DOI:** 10.3390/ijerph15091878

**Published:** 2018-08-30

**Authors:** Mark Yates, Jennifer J. Watts, Kasia Bail, Mohammadreza Mohebbi, Sean MacDermott, Jessica C. Jebramek, Henry Brodaty

**Affiliations:** 1 Ballarat Health Services, Deakin University, Faculty of Health, School of Medicine, Ballarat, VIC 3350, Australia; 2 School of Psychiatry, University of New South Wales, Sydney, NSW 2052, Australia; 3 Centre for Population Health Research, Faculty of Health, Deakin University, Burwood, VIC 3125, Australia; j.watts@deakin.edu.au; 4 Health Research Institute and Synergy Nursing and Midwifery Research Centre, University of Canberra, Canberra, ACT 2617, Australia; Kasia.Bail@canberra.edu.au; 5 Biostatistics Unit, Faculty of Health, Deakin University, Geelong, VIC 3220, Australia; m.mohebbi@deakin.edu.au; 6 La Trobe University, School of Rural Health, Mildura, VIC 3520, Australia; s.macdermott@latrobe.edu.au; 7 School of Psychology, Deakin University, Geelong, VIC 3220, Australia; jjebramek@deakin.edu.au; 8 Centre for Healthy Brain Ageing, Dementia Collaborative Research Centre, University of New South Wales, Sydney, NSW 2052, Australia; h.brodaty@unsw.edu.au

**Keywords:** delirium and cognitive disorders, acute hospital, nursing, nonclinical staff dementia, education, cost and cost analysis, system redesign

## Abstract

Despite the increasing number of older people, many with cognitive impairment (CI), in hospitals, there is yet to be an evaluation of hospital-wide interventions improving the management of those with CI. In hospitalized patients with CI, there are likely to be associations between increased complications that impact on outcomes, length of stay, and costs. This prospective study will evaluate the effectiveness of an established hospital CI support program on patient outcomes, patient quality of life, staff awareness of CI, and carer satisfaction. Using a stepped-wedge, continuous-recruitment method, the pre-intervention patient data will provide the control data for usual hospital care. The intervention, the Dementia Care in Hospitals Program, provides hospital-wide CI awareness and support education, and screening for all patients aged 65+, along with a bedside alert, the Cognitive Impairment Identifier. The primary outcome is a reduction in hospital-acquired complications: urinary tract infections, pressure injuries, pneumonia and delirium. Secondary outcome measures include cost effectiveness, patient quality of life, carer satisfaction, staff awareness of CI, and staff perceived impact of care. This large-sample study across four sites offers an opportunity for research evaluation of health service functioning at a whole-of-hospital level, which is important for sustainable change in hospital practice.

## 1. Introduction

Dementia is the leading cause of disability burden and the second leading cause of burden of disease for people aged 65+ [1]. Eighty-three-thousand patients with dementia are admitted to hospital each year in Australia [1].

People with dementia experience poorer outcomes of hospitalization, including higher rates of hospital-acquired complications [2]; higher rates of discharge to residential care [3]; higher morbidity [4]; and more bed days in hospital [5]. Outcome measurement is hampered by difficulty in relying on documentation of dementia diagnosis as it often goes undetected and, if detected, often goes undocumented [6].

In complex hospital environments, the diagnosis and documentation of dementia is made difficult by the presence of other causes of memory and thinking disorders, such as delirium, head injury, or narcosis. These disorders can be symptomatically similar to dementia regarding presentation of confusion and disorientation. The higher risks of hospital-acquired complications documented in people with dementia [2] could also be associated with these “failures in cognition”. Consequently, the term ‘cognitive impairment’ (CI) can be used to describe the memory and thinking difficulties seen in hospital without ascribing a diagnosis. CI is increasingly used as a term to encompass the breadth of disorders causing brain dysfunction, and in hospitals is most likely caused by dementia, delirium or stroke [7].

Adults admitted to Australian hospitals are assumed to have the cognitive capability to respond to the demands of hospital care, which require information retention, dexterity, insight and empathy. Some hospital safety systems, such as medication safety, rely on this cognitive ability in order to be effective [8]. It is estimated, however, that 30% of patients aged 65+ have CI during their hospital stay [7]. While dementia is the focus of much of the current research related to risks of hospitalization, this study will target patients with CI, who are likely to share similar high levels of risk.

Risk of hospitalization for patients with CI is compounded by the complexity of the hospital environment. Hospital staff are known to be inadequately equipped to identify, or respond appropriately to, people with dementia or other cognitive impairments [7]. Staff report difficulty in engaging with patients with CI and their families [9], and that older people’s needs may not align well with the acute hospital setting [10]. Patients with CI require higher support needs for communication, procedural interventions, ambulation, toileting, and medication administration [11]. Positive nursing work environments with manageable workloads [12,13,14], and acute models for the care of older people, have been associated with lower rates of hospital-acquired complications [15,16], indicating that, with suitable models of care, these are potentially modifiable patient outcomes.

Risk screening combined with appropriate mitigation processes on admission to hospital is a well-recognized approach to identify and reduce patient risk [17]. Similarly, screening for CI provides an opportunity for follow-up interventions and the implementation of appropriate care. Following up an effective screening program with a process for hospital-wide awareness of patients with CI, embedded by way of a simple staff–patient communication strategy, has the potential to reduce incidence of complications, improve hospital outcomes, reduce length of stay, and lower costs.

The Dementia Care in Hospitals Program (DCHP) provides an intervention that aims to change the hospital care paradigm from one where all hospitalized adults are expected to manage the complex demands of hospital care, to one where it is recognized that additional support and a change to the hospital processes and environment are needed for those with CI.

The DCHP is a hospital-based education and system-change program linked to screening for CI. Unlike dementia diagnosis, screening for CI does not require complex algorithms: it is a functional descriptor of impairment of memory and thinking associated with risk. Analogous to falls, the identification of this sort of functional risk can be embraced by nursing and allied health staff.

Previous evaluation of the program showed improvements in staff knowledge, confidence and comfort in dealing with patients with CI [9], and increased carer satisfaction [18]. The effect of the program on clinical outcomes, patient quality of life, and cost effectiveness has, however, not yet been measured.

### Study Aim and Hypotheses

The primary aim of this hospital-based interventional study is to determine if the DCHP will reduce hospital-acquired complications in people aged 65+ with CI, compared to those receiving usual hospital care. Secondary aims include an economic evaluation of the intervention compared to usual hospital care, and an examination of its impact on patient quality of life, carer satisfaction, staff awareness of CI, and staff perceived burden of care when caring for patients with CI in hospital.

## 2. Materials and Methods

### 2.1. Study Design and Setting

The impact evaluation of the DCHP will use a prospective, stepped-wedge, cross-sectional, continuous-recruitment study design. Patients aged 65+ will be screened for CI within 24 h of hospital admission and included if they have CI. Recruitment will occur continuously over the study period, with individual patients remaining in the study for the length of their hospital stay. Patients aged 65+ admitted to the target wards of a single hospital are referred to as a cluster; there will be four clusters across four Australian jurisdictions. Three sites are large metropolitan hospitals with a university affiliation; and one is a regional hospital. At each hospital, the wards selected to implement the DCHP will be determined by level of interest from the nursing leadership team and a requirement for a spread of wards across medical, surgical, and acute aged care. The Baseline period was determined by the estimated time required to achieve 80% patient screening and 40% staff education. The 42-week intervention period was considered sufficient time for change to be measurable within the constraints of the project funding. Consistent with a stepped-wedge trial design [19], each cluster provides both before and after observations, and clusters incrementally shift from control to intervention (see Table 1—Stepped-Wedge Timeline). Hospital-acquired complications data will be analyzed cross-sectionally.

### 2.2. Study Population

The participant pool will comprise all patients aged 65+ (50+ for those identifying as Aboriginal and Torres Strait Islander) admitted for more than 24 h to participating wards and screened for CI using a validated screening tool. There will be no patient exclusions. Patients transferred between participating wards will be counted once. Patients transferred to a participating ward from a nonparticipating ward will be included if they stay on the participating ward for longer than 24 h, and patients transferred from a participating ward on which they had stayed for greater than 24 h to a nonparticipating ward will also be included. Consent was confirmed with each participating health service via waiver of consent documentation.

The Dementia Quality of Life Measure (DemQol) and Carer surveys will be completed by a subset of the participant pool who screen positive for CI, and their carers. Staff surveys will be completed by personnel on the relevant wards who have regular contact with the target patient group. These will include clinical (nursing, medical and allied health) and nonclinical staff (ward clerks, food and domestic staff, porters and cleaners) on the participating wards.

### 2.3. Materials

The choice of screening tool was determined by the hospitals at the time of site selection. It was requested that a validated screening tool be used, and hospitals chose tools that were already embedded in current practice. Criteria and screening tools for CI and study inclusion are presented in Table 2.

Each hospital will receive face-to-face and remote support from the DCHP National Implementation Team based at Ballarat Health Services. Project implementation will support a full-time on-site project officer, materials, and travel funding. The implementation team will visit each site for a project launch and education session. Local education will be supported by the project officer and provision of an education package that is appropriate for both clinical and nonclinical staff.

### 2.4. Data

Hospital-acquired complication data will be drawn from routinely collected hospital data sets and submitted to the Health Roundtable (HRT) [24], which is a not-for-profit membership organization of participating hospitals across Australia and New Zealand. HRT holds routinely collected data for 8 years for all the hospitals in the DCHP. The hospital-acquired complications for the evaluation were derived from hospital-coded patient records. This method is based on work by Needleman et al. [12], and updated to ICD10 by Bail et al. [2]. All the diagnostic codes for the patients’ episodes will be examined separately. Patients with at least one of four complications will be recorded as positive for a hospital-acquired complication.

Staff surveys, carer surveys and patient DemQoLs [25,26] will be collected during the pre-intervention period and at approximately six months post-intervention.

Ethics approval has been obtained for data sharing with the external evaluation team. Patient data, which will be de-identified prior to analysis, will include patient descriptors (e.g., age, name, Unit Record Number (UR), Aboriginal and Torres Strait Islander (ATSI) status, comorbidity index), and episode descriptors (e.g., length of stay, discharge ward).

Data will be stored on a secure server with restricted access. Data will be entered into Excel spreadsheets on-site and at the DCHP National Office for later analysis using the SPSS and Stata statistical software packages. To ensure data integrity, all data cleaning and data entry will be completed by two research assistants using a cross-check methodology. Responsibility for appropriate and secure data storage rests with the primary investigator and the evaluation team.

### 2.5. Outcome Measures

The primary outcome measure will be the change in the rate of the combined risk of one or more of the following hospital-acquired complications: urinary tract infection, pressure injury, pneumonia or delirium before and after the adoption of the DCHP. The rate of this combined risk will be determined for patients who screen positive for CI over a 12-week period before the introduction of the DCHP, and for patients who screen positive for CI over four consecutive 12-week periods during implementation. A summary of primary and secondary outcomes as well as relevant instruments and sources is provided in Table 3. Falls rates will be a secondary outcome as per Medibank Private methodology, using the Y9222 flag within the ICD code to indicate whether a fall occurred in hospital or not [27].

### 2.6. Intervention

#### 2.6.1. DCHP Implementation, Hospital Engagement and Ward Delivery

**Figure 1:**
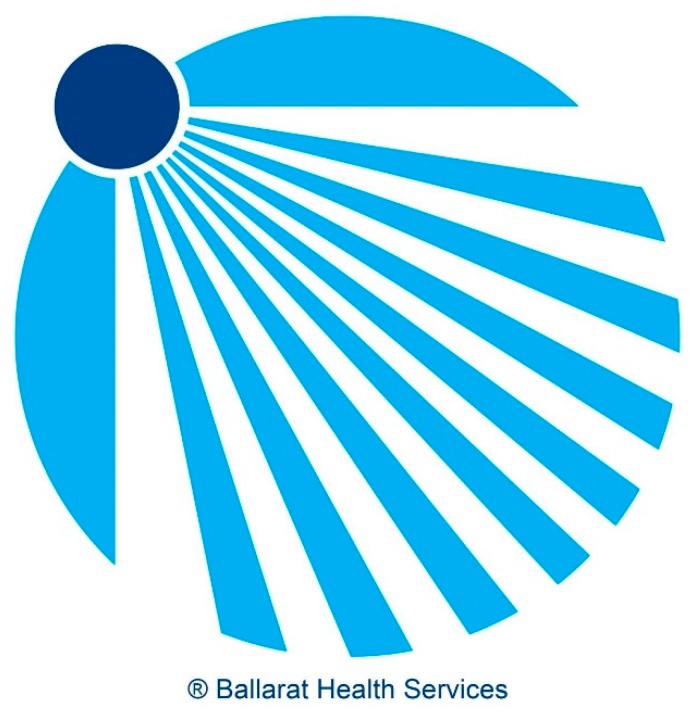
Cognitive Impairment Identifier (CII).

The DCHP includes a whole-of-hospital communication training program for all staff. Other key components are the development of hospital-specific clinical pathways (protocols, policies), executive management support, and ward-based ‘champions’ for the identification and management of CI. The DCHP also requires close engagement with carers/family of patients with CI. The primary vehicle for the program’s hospital engagement is an easily recognizable bedside alert: the ‘Cognitive Impairment Identifier’ (CII—see Figure 1).

The education and training sessions are aimed at enabling staff to better understand, and work with, patients with CI and their carers/family. Initial training of the local teaching staff will be conducted by the DCHP National Implementation Team. It is anticipated that, during implementation, the education program will be delivered to 80% of all staff with patient contact on target wards. Cross-over from Baseline to Intervention will not occur until a minimum of 40% of target staff have received training.

#### 2.6.2. Measures of Successful Implementation and Confounders

Successful adoption of the DCHP will have significant influence on the results of the evaluation. Target ward screening rates will be collected monthly. It is anticipated that 100% of patients aged 65+ will be screened and the CII used for 80% of those who screen positively. Staff education rates will be monitored to ensure a sustained rate of 80% over the 12 months of the program.

#### 2.6.3. Intervention Costs

The resources used in the intervention will be fully costed and attributed to the inpatient population aged 65+ in the relevant wards. This includes the cost of developing the training package, staff training costs (time), and the time taken to conduct screening. The cost of the training package will be treated as a capital cost with an allowance made for periodic updates. Costs attributable to evaluation will not be included in the cost analysis.

#### 2.6.4. Blinding

Blinding will not occur, as there is no practical way to blind hospital staff or members of the evaluation teams.

#### 2.6.5. Sample Size, Power Calculation and Analysis

Based on a conservative estimate of 750 positive screens for CI in each period, the target sample size is a total of 3750 patients. The required sample has been calculated with power 0.8, type I error 0.05 and design effects based on clustering effect (ICC) at site (0.05) level. It is estimated that for a complication rate ranging from 20–26%, this sample size will enable detection of a 22–25.5% or more reduction in risk ratio (RR). This is equivalent to a minimum 5–5.5% reduction in absolute risk. A generalized linear mixed model for a binary outcome with time epoch as a fixed factor and hospitals as a random effect for an incomplete stepped-wedge design with a cross-sectional design was adopted for the power calculation [29]. Stata software was used for this purpose [30].

In relation to the secondary outcomes, based on type I error of 0.05 and a power of 0.9, a target sample size of 160 DemQols per site (80 at baseline and 80 at intervention) is required to detect 15-point differences in health-related quality-of-life score.

Assuming a type I error of 0.05 and a power of 0.8, a target sample size of 140 carer surveys (70 at baseline and 70 at intervention) is required to detect a minimum effect size of 0.4.

#### 2.6.6. Ethics Approval

The study protocol has been approved by the relevant Ethics Committee at each site. Ethics approval numbers are as follows: HREC/15/TQEH/9 (Government of South Australia, SA Health, Human Research Ethics Committee); ETH.6.15.105 (ACT Health, Human Research Ethics Committee); HREC/15/TQEH/9 (Human Research Ethics Committee (Tasmania) Network); 2015-103 (Government of Western Australia, Department of Health, Human Research Ethics Committee).

The Registration Number is Australian New Zealand Clinical Trials Registry (ANZCTR) ACTRN12615000905561.

## 3. Discussion

A stepped-wedge design is a logical fit, given that hospitals are constantly changing their care models in different ways and across varying time courses. The stepped-wedge methodology is designed to control for potential bias that occurs as a result of variations in hospital practice and the introduction of new regulations. In addition, while the value of the program has been accepted by the funding body and participating sites on the basis of previous rollouts and grey literature reports [9,18], this study is expected to provide a platform for adoption of the DCHP across each State in Australia. Such an outcome requires robust evidence of clinical benefit and cost effectiveness, particularly as Australia is a federated democracy where acute hospital care is the responsibility of individual States.

The order of implementation is not randomized. While randomization would be preferable, with only four sites this is not critical. In addition, introducing significant change in hospitals requires hospital ownership of the program, which is facilitated by a negotiated start date reflecting organizational readiness.

This evaluation will measure the hospital-acquired complication rate in a large prospective cohort of older Australian hospital patients with and without CI. The international literature on hospital-acquired complication rates in patients with dementia has been based on retrospective data [31], which is known to be incomplete and potentially missing half the at-risk group [3]. New international studies are prospective but smaller in terms of sample sizes [32]. Real-time capture of data on all older patients with CI in hospital may have the effect of either decreasing or increasing the recorded hospital-acquired complication rate. We hypothesize that patients with mild symptoms, who are possibly missed in retrospective studies, are at greater risk because they receive no or little support beyond that given for their documented comorbidities. Therefore the risk rate will be higher. It is possible that this assumption is incorrect and the complications are rather a reflection of the degree of CI, resulting in a reduced complication rate reflecting milder CI.

As CI, which may include delirium, is the inclusion criterion, and delirium is an outcome measure, there is a risk that the impact of the DCHP on delirium may be confounded. Delirium, as an outcome measure in this study, would require staff to have not recorded it as a problem when the CI screening was completed, but to have recorded it at a later stage. As this would suggest an escalation in the symptomology on at least a behavioral level, a factor on which the DCHP impacts, it was decided that the retention of delirium as an outcome measure is methodologically sound.

The impact of the DCHP on hospital-acquired complications will be influenced by how successfully the program is embedded into the hospital system. Measurement of changing carer satisfaction, change in staff practice and awareness, and patient quality of life will act as surrogate markers of individual hospital program adoption.

## 4. Limitations

The CI screening tools will not be consistent across all sites. It is possible, therefore, that the population identified as at risk of hospital-acquired complications will vary across participating hospitals. This variation will be mitigated by pooling the results across all four sites. The sensitivity and specificity values of the screening tools used at the four sites are similar (AMT4 ; sensitivity 80% and specificity 76% [33]; AMTS; sensitivity 81% specificity 84% [34]; MiniCog; sensitivity 76% and specificity 73% [35]). The Clock Drawing Test [23] was used in addition to some screening tools as it has previously been found to increase the sensitivity and specificity of other screening tests [36]. As there are a wide variety of CI screening tools used in Australia, the variance in tool use reflects real practice.

Equally, while there is relative consistency of data extraction methodology, in Australia there is variance in how hospital events are recorded. This may also impact on the final data.

The target number of positive screens for CI was estimated from national data and experience from previous rollouts of the DCHP in Victoria. If the number of positive screens is significantly fewer than the predicted number, this could impact on the statistical power of the study.

While there were no exclusions within the target recruitment population, it is likely that universal screening of all patients 65+ in a hospital environment will not be achieved. The results will be reported on an intention-to-treat basis. This is a conservative approach but is likely to reflect real-world practice, so a positive result is more likely to translate across hospitals.

The DEMQoL [25,26] was not originally designed for use in the hospital setting. It is expected that there may be a significant number of patients who may not be able to complete the survey because of ill-health.

The National Commission on Safety and Quality in Health Care has released advice relating to screening for CI in acute care, and the screening for, and management of, delirium (ACSQHC 2013). As this advice will be taken up at the state level gradually, there may be some impact on screening practice that is not attributable to implementation of the DCHP which should be mitigated by the study design.

## 5. Conclusions

This is the first large multi-site prospective trial to evaluate the impact of a program to detect and manage cognitive impairment in the acute hospital setting.

## 6. Patents

The CII is a registered trademark held by Ballarat Health Services. This has been done to protect its integrity and appropriate usage. Licensing is free to Australian health services.

## Figures and Tables

**Table 1:** Stepped-Wedge Timeline.

Timeline	February 2014 to February 2015	March 2015	June 2015	September 2015	December 2015	March 2016	May 2016	July 2016	September 2016	December 2016	February 2017
Weeks	52	12	12	12	12	12	12	12	12	12	12
1	BL1 Normal Practice	BL1 Normal Practice	**BL2 Training**	**BL2 Training**	T1	T2	T3	T4			T5
2	BL1 Normal Practice	BL1 Normal Practice	BL1 Normal Practice	**BL2 Training**	**BL2 Training**	T1	T2	T3	T4		T5
3	BL1 Normal Practice	BL1 Normal Practice	BL1 Normal Practice	BL1 Normal Practice	BL1 Normal Practice	**BL2 Training**	T1	T2	T3	T4	T5
4	BL1 Normal Practice	BL1 Normal Practice	BL1 Normal Practice	BL1 Normal Practice	BL1 Normal Practice	BL1 Normal Practice	**BL2 Training**	T1	T2	T3	T4

BL1 = Normal Practice; BL2 = Baseline / Training Phase (yellow); T1–T5 = Intervention phases (green).

**Table 2:** Cognitive Impairment Screening Tools.

Tool	Abbreviation	Criteria for Positive CI Screen	Used by Site	Reference
Abbreviated Mental Test	AMT	Score≤ 7	1	Hodkinson [20]
Mini-Cog		Recall 1 or 2 of 3 items and abnormal Clock Drawing; or recall of 0 of 3 words.	4	Borson [21]
Abbreviated Mental Test Score 4 *	AMT4	Score of 3 or less	2 and 3	Swain [22]
Clock Drawing Test ^	CDT	Not all clock numbers present, spaced unevenly, or hands pointing to incorrect time.	2, 3 and 4	Scanlan [23]

* Only used in conjunction with Clock Drawing Test (CDT); ^ used in conjunction with either AMT4 or MiniCog.

**Table 3:** Outcome measure, instruments and data sources.

	Outcome Measure	Instrument/Source
**Primary Outcome: Clinical**	The change in the rate of the combined risk of one or more of the hospital-acquired complications: urinary tract infection, pressure injury, pneumonia and delirium, before and after the adoption of the DCHP.	Health Roundtable [24]
**Primary Outcome: Cost**	Difference in mean total cost of hospitalization for patients aged 65+ before and after the adoption of the DCHP. Difference in mean total cost of hospitalization for patients aged 65+ who screen positive for CI before and after the adoption of the DCHP.	Health Roundtable [24]
**Secondary Outcome: Cost Effectiveness**	Quality of life Length of stay Cost effectiveness	DemQol ^ [25,26] Health Roundtable [24]
**Secondary Outcome: Satisfaction/Confidence**	Staff knowledge and confidence Carer satisfaction	Staff survey # (see Supplementary Materials S1 & S3) Carer survey # (see Supplementary Materials S2 & S4)
**Secondary Outcome: Medication and Patient Safety**	Total antipsychotic usage per admission Anticholinergic load per admission [28] In-hospital falls and mortality	Medical Record review Health Roundtable [24]
**Secondary Outcome: Sustainability**	The rate of the combined risk of one or more of: urinary tract infection, pressure injury, pneumonia and delirium in patients 65+ with CI occurring during the hospital admission.	Health Roundtable [24]

# Both surveys have been developed for the Dementia Care in Hospitals Program (DCHP, https://www.bhs.org.au/node/130) and used across all 25 rollouts. The questions target key education goals including changes to staff attitudes and actions. Attached as Supplementary Materials; ^ DemQol [25,26] will be used for patients who screen positive for Cognitive Impairment (CI).

## Supplementary Materials

The following are available online at http://www.mdpi.com/1660-4601/15/9/1878/s1.

## References

[1] Australian Institute of Health and Welfare (2012). Dementia in Australia.

[2] Bail K., Berry H., Grealish L., Draper B., Karmel R., Gibson D., Peut A. (2013). Potentially preventable complications of urinary tract infections, pressure areas, pneumonia, and delirium in hospitalised dementia patients: Retrospective cohort study. BMJ Open.

[3] Draper B., Karmel R., Gibson D., Peut A., Anderson P. (2011). The hospital dementia services project: Age differences in hospital stays for older people with and without dementia. Int. Psychogeriatr..

[4] Sampson E.L., Blanchard M.R., Jones L., Tookman A., King M. (2009). Dementia in the acute hospital: Prospective cohort study of prevalence and mortality. Br. J. Psychiatry.

[5] Bail K., Goss J., Draper B., Berry H., Karmel R., Gibson D. (2015). The cost of hospital-acquired complications for older people with and without dementia; a retrospective cohort study. BMC Health Serv. Res..

[6] Laurila J.V., Pitkala K.H., Strandberg T.E., Tilvis R.S. (2004). Detection and documentation of dementia and delirium in acute geriatric wards. Gen. Hosp. Psychiatry.

[7] Australian Commission on Safety and Quality in Health Care Evidence for the Safety and Quality Issues Associated with the Care of Patients with Cognitive Impairment in Acute Settings: A Rapid Review. https://www.safetyandquality.gov.au/publications.

[8] American Hospital Association, Health Research and Educational Trust, the Institute for Safe Medication Practices (2002). Pathways to Medication Safety: Looking Collectively at Risk: Chicago. https://psnet.ahrq.gov/resources/resource/1336/looking-collectively-at-risk.

[9] Foreman P., Gardner I. (2007). Evaluation of Education and Training of Staff in Dementia Care and Management in Acute Settings.

[10] Skirbekk H., Nortvedt P. (2014). Inadequate treatment for elderly patients: Professional norms and tight budgets could cause “ageism” in hospitals. Health Care Anal..

[11] Bail K., Grealish L. (2016). ‘Failure to maintain’: A theoretical proposition for a new quality indicator of nurse care rationing for complex older people in hospital. Int. J. Nurs. Stud..

[12] Needleman J., Buerhaus P., Potter V., Mattke S., Stewart M., Zelevinsky K. (2001). Nurse Staffing and Patient Outcomes in Hospitals.

[13] Cimiotti J.P., Aiken L.H., Sloane D.M., Wu E.S. (2012). Nurse staffing, burnout, and health care-associated infection. Am. J. Infect. Control.

[14] Twigg D., Duffield C., Bremner A., Rapley P., Finn J. (2011). The impact of the nursing hours per patient day (NHPPD) staffing method on patient outcomes: A retrospective analysis of patient and staffing data. Int. J. Nurs. Stud..

[15] Chong M.S., Chan M., Tay L., Ding Y.Y. (2014). Outcomes of an innovative model of acute delirium care: The Geriatric Monitoring Unit (GMU). Clin. Interv. Aging.

[16] Fox M.T., Persaud M., Maimets I., O’brien K., Brooks D., Tregunno D., Schraa E. (2012). Effectiveness of acute geriatric unit care using acute care for elders components: A systematic review and meta-analysis. J. Am. Geriatr. Soc..

[17] Lester W., Freemantle N., Begaj I., Ray D., Wood J., Pagano D. (2013). Fatal venous thromboembolism associated with hospital admission: A cohort study to assess the impact of a national risk assessment target. Heart.

[18] Theobald M., Yates M., McIntyre I. Cognitive Impairment Identifier Project—An All of Hospital Education Program to Improve the Awareness of and Communication with People with Dementia—Linked to a Visual Cognitive Impairment Identifier. https://www.bhs.org.au/dchp.

[19] Hemming K., Lilford R., Girling A.J. (2015). Stepped-wedge cluster randomised controlled trials: A generic framework including parallel and multiple-level designs. Stat. Med..

[20] Hodkinson H.M. (2012). Evaluation of a mental test score for assessment of mental impairment in the elderly. Age Ageing.

[21] Borson S., Scanlan J.M., Watanabe J., Tu S.-P., Lessig M. (2006). Improving identification of cognitive impairment in primary care. Int. J. Geriatr. Psychiatry.

[22] Swain D.G., Nightingale P.G. (1997). Evaluation of a shortened version of the abbreviated mental test in a series of elderly patients. Clin. Rehabil..

[23] Scanlan J.M., Brush M., Quijano C., Borson S. (2002). Comparing clock tests for dementia screening: Naïve judgments vs formal systems—What is optimal?. Int. J. Geriatr. Psychiatry.

[24] The Health Roundtable—Promoting Innovation in Healthcare. https://www.healthroundtable.org/.

[25] Smith S.C., Lamping D.L., Banerjee S., Harwood R.H., Foley B., Smith P., Cook J.C., Murray J., Prince M., Levin E. (2007). Development of a new measure of health-related quality of life for people with dementia: Demqol. Psychol. Med..

[26] Rowen D., Mulhern B., Banerjee S., van Hout B., Young T.A., Knapp M., Smith S.C., Lamping D.L., Brazier J.E. (2012). Estimating preference-based single index measures for dementia using demqol and demqol-proxy. Value Health.

[27] Medibank Private Health Hospital Acquired Complications. https://www.medibank.com.au/content/dam/medibank/calvary/medibank_hospital_acquired_complications_list_2015.pdf.

[28] Rudolph J.L., Salow M.J., Angelini M.C., McGlinchey R.E. (2008). The anticholinergic risk scale and anticholinergic adverse effects in older persons. Arch. Intern. Med..

[29] Hussey M.A., Hughes J.P. (2007). Design and analysis of stepped-wedge cluster randomized trials. Contemp. Clin. Trials.

[30] Hemming K., Girling A. (2014). A menu-driven facility for power and detectable-difference calculations in stepped-wedge cluster-randomized trials. Stata J..

[31] Sari A.B.A., Cracknell A., Sheldon T.A. (2008). Incidence, preventability and consequences of adverse events in older people: Results of a retrospective case-note review. Age Ageing.

[32] Szlejf C., Farfel J.M., Curiati J.A., Couto E.D., Jacob W., Azevedo R.S. (2012). Medical adverse events in elderly hospitalized patients: A prospective study. Clinics.

[33] Schofield I., Stott D.J., Tolson D., McFadyen A., Monaghan J., Nelson D. (2010). Screening for cognitive impairment in older people attending accident and emergency using the 4-item abbreviated mental test. Eur. J. Emerg. Med..

[34] Incalzi R.A., Cesari M., Pedone C., Carosella L., Carbonin P.U. (2003). Construct validity of the abbreviated mental test in older medical inpatients. Dement. Geriatr. Cognit. Disord..

[35] Holsinger T., Plassman B.L., Stechuchak K.M., Burke J.R., Coffman C.J., Williams J.J. (2012). Screening for cognitive impairment: Comparing the performance of four instruments in primary care. J. Am. Geriatr. Soc..

[36] Mittal C., Gorthi S.P., Rohatgi S. (2010). Early cognitive impairment: Role of clock drawing test. Med. J. Armed Forces India.

