# Supplementary material for: Evaluating the Impact of the Dementia Care in Hospitals Program (DCHP) on Hospital-Acquired Complications: Study Protocol
Source: Int J Environ Res Public Health. 2018 Aug 30;15(9):1878. doi: 10.3390/ijerph15091878 (PMC6165270; doi:10.3390/ijerph15091878)
Supplement: Supplementary file 1 [file ijerph-15-01878-s001.zip › Supplementary/A1 Carer satisfaction baseline V2.pdf]

Dementia Care in Hospitals Program

Carer Satisfaction Survey - Baseline

Please circle the number which best represents your opinion:

1. How satisfied are you the hospital staff knew the person you care for has problems with memory and thinking?

**1**	**2**	**3**	**4**	**5**	**?**
Very Dissatisfied	Dissatisfied	Neither	Satisfied	Very Satisfied	Unsure

2. How satisfied are you staff introduced themselves to the person you care for on a regular basis?

**1**	**2**	**3**	**4**	**5**	**?**
Very Dissatisfied	Dissatisfied	Neither	Satisfied	Very Satisfied	Unsure

3. Are you satisfied the person you care for was not expected to do more than they were capable of? e.g. remembering to keep to a fluid restriction, attending to toileting needs etc.

**1**	**2**	**3**	**4**	**5**	**?**
Very Dissatisfied	Dissatisfied	Neither	Satisfied	Very Satisfied	Unsure

4. Are you satisfied the staff explained things to the person you care for in a simple way and checked if they were understood?

**1**	**2**	**3**	**4**	**5**	**?**
Very Dissatisfied	Dissatisfied	Neither	Satisfied	Very Satisfied	Unsure

5. How satisfied are you the staff made you welcome to provide information about the person you care for? e.g. were you asked about their likes and dislikes, or difficulties they have with communication?

**1**	**2**	**3**	**4**	**5**	**?**
Very Dissatisfied	Dissatisfied	Neither	Satisfied	Very Satisfied	Unsure

6. When you voluntarily offered information regarding the person you care for, how satisfied are you the staff listened to or took notice of you?

**1**	**2**	**3**	**4**	**5**	**?**
Very Dissatisfied	Dissatisfied	Neither	Satisfied	Very Satisfied	Unsure

7. Are you satisfied the hospital staff was understanding of any challenging behaviours that may have been exhibited by the person you care for?

**1**	**2**	**3**	**4**	**5**	**?**
Very Dissatisfied	Dissatisfied	Neither	Satisfied	Very Satisfied	Unsure

8. Have you and /or the person you care for had positive experiences within a hospital setting?

Yes/No?

If yes, what made it positive?

If no, what made it negative?

9.How satisfied are you with the information you were given about the condition and treatment of the person you care for?

1	2	3	4	5	?
Very Dissatisfied	Dissatisfied	Neither	Satisfied	Very Satisfied	Unsure

10. How satisfied are you hospital staff gave you the option of receiving discharge information for the person you care for? e.g. information about follow-up appointments, medication changes

1	2	3	4	5	?
Very Dissatisfied	Dissatisfied	Neither	Satisfied	Very Satisfied	Unsure

If satisfied was it adequate?

Yes/No?

11. How satisfied are you this hospital is friendly for people with memory and thinking difficulties and their carers?

1	2	3	4	5	?
Very Dissatisfied	Dissatisfied	Neither	Satisfied	Very Satisfied	Unsure

All feedback is very useful for us in better meeting the needs of people with memory and thinking difficulties.
Please feel free to make any additional comments regarding our care for people with memory and thinking difficulties.

Should you have any questions regarding this questionnaire contact?

Fill in contact here……….

Thank you for your time and effort in completing this survey.
