# Supplementary material for: Evaluating the Impact of the Dementia Care in Hospitals Program (DCHP) on Hospital-Acquired Complications: Study Protocol
Source: Int J Environ Res Public Health. 2018 Aug 30;15(9):1878. doi: 10.3390/ijerph15091878 (PMC6165270; doi:10.3390/ijerph15091878)
Supplement: Supplementary file 1 [file ijerph-15-01878-s001.zip › Supplementary/A3 Staff education survey - Pre.pdf]

Dementia Care in Hospitals Program

Staff Education Survey – Pre DCHP

Prior to the education program on dementia care in hospitals, we are interested in your views and experience of dealing with patients with cognitive impairment and their carer/family. The information will assist the hospital in training staff, improving the quality of care for these patients, and improving communication with carers/families.

All replies will be strictly confidential and you will not be identified in any way.

Please circle the response that best describes your position.

Clinical staff, e g nursing, medical. allied health etc. **Non-clinical staff** e g engineers. ward clerks. etc

Non-clinical Staff: Have you ever been offered in-service or education on dementia or delirium?

Yes No

1. **What proportion of patients do you think you come across in the hospital with dementia, delirium or memory and thinking difficulties?**

10% 20% 30% 40% 50% 60% 70% 80% 90%

2. **How would you rate your confidence in dealing with patients with dementia, delirium or memory and thinking difficulties?**

very low low satisfactory high very high

3. **How would you rate your level of comfort in dealing with patients with dementia, delirium or memory and thinking difficulties?**

very low low satisfactory high very high

4. **How would you rate the level of organisational support you receive \\/\\/hen dealing with patients with dementia, delirium or memory and thinking difficulties?**

very low low satisfactory high very high

5. **How would you rate your level of job satisfaction in dealing with patients with dementia, delirium or memory and thinking difficulties?**

very low | low | satisfactory | high | very high

6. **In your experience how well equipped is the hospital environment to meet the needs of patients with dementia, delirium or memory and thinking difficulties?**

very low | low | satisfactory | high | very high
