# Supplementary material for: Evaluating the Impact of the Dementia Care in Hospitals Program (DCHP) on Hospital-Acquired Complications: Study Protocol
Source: Int J Environ Res Public Health. 2018 Aug 30;15(9):1878. doi: 10.3390/ijerph15091878 (PMC6165270; doi:10.3390/ijerph15091878)
Supplement: Supplementary file 1 [file ijerph-15-01878-s001.zip › Supplementary/A4 Staff Education Survey post.pdf]

Dementia Care in Hospitals Program

Staff Education Survey – Post DCHP

You will have completed a questionnaire prior to receiving education about the Cognitive Impairment Identifier Project. We are interested in your views and experience in dealing with patients with cognitive impairment and their carer's/families since receiving this education. The information gathered from the attached questionnaire will assist the hospital in training staff, improving the quality of care for these patients, and improving communication with carers/families.

All replies will be strictly confidential and you will not be identified in any way.

Please circle the response that best describes your role

Clinical staff e g nursing, medical. allied health etc. **Non-clinical** e g engineers. ward clerks. etc

All participants please circle the response that best reflects your experience.

1. **What proportion of patients do you think you come across in the hospital with dementia, delirium or memory and thinking difficulties?**

   10% 20% 30% 40% 50% 60% 70% 80% 90%

2. **How would you rate your confidence in dealing with patients with dementia, delirium or memory and thinking difficulties?**

   very low low satisfactory high very high

3. **How would you rate your level of comfort in dealing with patients with dementia, delirium or memory and thinking difficulties?**

   very low low satisfactory high very high

4. **How would you rate the level of organisational support you receive when dealing with patients with dementia, delirium or memory and thinking difficulties?**

   very low low satisfactory high very high

5. How would you rate your level of job satisfaction in dealing with patients with dementia, delirium or memory and thinking difficulties?

very low low satisfactory high very high

6. In your experience how well equipped is the hospital environment to meet the needs of patients with dementia, delirium or memory and thinking difficulties?

very low low satisfactory high very high
