# Supplementary material for: Evaluating the Impact of the Dementia Care in Hospitals Program (DCHP) on Hospital-Acquired Complications: Study Protocol
Source: Int J Environ Res Public Health. 2018 Aug 30;15(9):1878. doi: 10.3390/ijerph15091878 (PMC6165270; doi:10.3390/ijerph15091878)
Supplement: Supplementary file 1 [file ijerph-15-01878-s001.zip › Supplementary/A5 DEMQOL Pre-Implementation in Word 0.1.pdf]

DCHP Post-Implementation

Final field test item-reduced DEMQOL (v4)

Study ID | | | | | | | | | |

DEMQOL (version 4)
To be used with interviewer manual

1. Ensure you have permission from ward/unit clinical staff to administer this survey to this patient
2. Ensure that you have provided the patient with a large print copy of the DCHP patient information sheet
3. Ensure that you have read out aloud to the patient the following (**in bold**)

This hospital is planning to introduce the 'Dementia Care in Hospitals Program'. You are being asked to complete a survey to help us understand your sense of wellbeing now. This will help us better understand if the Dementia Care in Hospitals Program when introduced improves the wellbeing of patients. The survey will take between 5-10 minutes to complete. You are free to choose whether to complete the survey. Should you choose not to complete the survey your care will not be compromised in any way. By completing this survey you agree to the research team collecting and using the information you have provided for the research project. Any information obtained in connection with this research project that can identify you will remain confidential. Your information will only be used for the purpose of this research project.

Instructions: Read each of the following questions (**in bold**) verbatim and show the respondent the response card.

I would like to ask you about your life. There are no right or wrong answers. Just give the answer that best describes how you have felt in the last week. Don't worry if some questions appear not to apply to you. We have asked the same questions of everybody.

Before we start we'll do a practice question; that's one that doesn't count. *(Show the response card and ask the respondent to say or point to the answer)*. **In the last week, how much have you enjoyed watching television?**

a lot **quite a bit** **a little** **not at all**

Follow up with a prompt question: **Why is that?** *or* **Tell me a bit more than that.**

For all of the questions I'm going to ask you, I want you to think about the last week.

First I'm going to ask about <u>your feelings</u>. In the last week, have you felt …

1.	**cheerful? ****	☐ **a lot**	☐ **quite a bit**	☐ **a little**	☐ **not at all**
2.	**worried or anxious?**	☐ **a lot**	☐ **quite a bit**	☐ **a little**	☐ **not at all**
3.	**that you are enjoying life?****	☐ **a lot**	☐ **quite a bit**	☐ **a little**	☐ **not at all**
4.	**frustrated?**	☐ **a lot**	☐ **quite a bit**	☐ **a little**	☐ **not at all**
5.	**confident?****	☐ **a lot**	☐ **quite a bit**	☐ **a little**	☐ **not at all**
6.	**full of energy?****	☐ **a lot**	☐ **quite a bit**	☐ **a little**	☐ **not at all**
7.	**sad?**	☐ **a lot**	☐ **quite a bit**	☐ **a little**	☐ **not at all**
8.	**lonely?**	☐ **a lot**	☐ **quite a bit**	☐ **a little**	☐ **not at all**
9.	**distressed?**	☐ **a lot**	☐ **quite a bit**	☐ **a little**	☐ **not at all**
10.	**lively?****	☐ **a lot**	☐ **quite a bit**	☐ **a little**	☐ **not at all**
11.	**irritable?**	☐ **a lot**	☐ **quite a bit**	☐ **a little**	☐ **not at all**
12.	**fed-up?**	☐ **a lot**	☐ **quite a bit**	☐ **a little**	☐ **not at all**
13.	**that there are things that you wanted to do but couldn't?**	☐ **a lot**	☐ **quite a bit**	☐ **a little**	☐ **not at all**

Next, I'm going to ask you about <u>your memory</u>. In the last week, how worried have you been about …

14.	**forgetting things that happened recently?**	☐ **a lot**	☐ **quite a bit**	☐ **a little**	☐ **not at all**
15.	**forgetting who people are?**	☐ **a lot**	☐ **quite a bit**	☐ **a little**	☐ **not at all**
16.	**forgetting what day it is?**	☐ **a lot**	☐ **quite a bit**	☐ **a little**	☐ **not at all**
17.	**your thoughts being muddled?**	☐ **a lot**	☐ **quite a bit**	☐ **a little**	☐ **not at all**
18.	**difficulty making decisions?**	☐ **a lot**	☐ **quite a bit**	☐ **a little**	☐ **not at all**
19.	**poor concentration?**	☐ **a lot**	☐ **quite a bit**	☐ **a little**	☐ **not at all**

Now, I'm going to ask you about your everyday life. In the last week, how worried have you been about …

20.	**not having enough company?**	☐ **a lot**	☐ **quite a bit**	☐ **a little**	☐ **not at all**
21.	**how you get on with people close to you?**	☐ **a lot**	☐ **quite a bit**	☐ **a little**	☐ **not at all**
22.	**getting the affection that you want?**	☐ **a lot**	☐ **quite a bit**	☐ **a little**	☐ **not at all**
23.	**people not listening to you?**	☐ **a lot**	☐ **quite a bit**	☐ **a little**	☐ **not at all**
24.	**making yourself understood?**	☐ **a lot**	☐ **quite a bit**	☐ **a little**	☐ **not at all**
25.	**getting help when you need it?**	☐ **a lot**	☐ **quite a bit**	☐ **a little**	☐ **not at all**
26.	**getting to the toilet in time?**	☐ **a lot**	☐ **quite a bit**	☐ **a little**	☐ **not at all**
27.	**how you feel in yourself?**	☐ **a lot**	☐ **quite a bit**	☐ **a little**	☐ **not at all**
28.	**your health overall?**	☐ **a lot**	☐ **quite a bit**	☐ **a little**	☐ **not at all**

We've already talked about lots of things: your feelings, memory and everday life. Thinking about all these things in the last week, how would you rate …

29.	**your quality of life overall?**?**	☐ **very good**	☐ **good**	☐ **fair**	☐ **poor**

**items that need to be reversed before scoring
